# Plant Source Derived Compound Exhibited *In Silico* Inhibition of Membrane Glycoprotein In SARS-CoV-2: Paving the Way to Discover a New Class of Compound For Treatment of COVID-19

**DOI:** 10.3389/fphar.2022.805344

**Published:** 2022-04-07

**Authors:** Saurov Mahanta, Tufan Naiya, Kunal Biswas, Liza Changkakoti, Yugal Kishore Mohanta, Bhaben Tanti, Awdhesh Kumar Mishra, Tapan Kumar Mohanta, Nanaocha Sharma

**Affiliations:** ^1^ National Institute of Electronics and Information Technology (NIELIT), Guwahati, India; ^2^ Department of Biotechnology, Maulana Abul Kalam Azad University of Technology, West Bengal, India; ^3^ Centre for Nanoscience and Nanotechnology, Sathyabama Institute of Science and Technology, Chennai, India; ^4^ Department of Applied Biology, School of Biological Sciences, University of Science and Technology Meghalaya (USTM), Baridua, India; ^5^ Department of Botany, Gauhati University, Guwahati, India; ^6^ Department of Biotechnology, Yeungnam University, Gyeongsan, South Korea; ^7^ Natural and Medical Sciences Research Centre, University of Nizwa, Nizwa, Oman; ^8^ Institute of Bioresources and Sustainable Development, Imphal, India

**Keywords:** homology modelling, phyto-compound, molecular dynamics, SARS-CoV2, *in silico*, membrane glycoprotein

## Abstract

SARS-CoV-2 is the virus responsible for causing COVID-19 disease in humans, creating the recent pandemic across the world, where lower production of Type I Interferon (IFN-I) is associated with the deadly form of the disease. Membrane protein or SARS-CoV-2 M proteins are known to be the major reason behind the lower production of human IFN-I by suppressing the expression of IFNβ and Interferon Stimulated Genes. In this study, 7,832 compounds from 32 medicinal plants of India possessing traditional knowledge linkage with pneumonia-like disease treatment, were screened against the Homology-Modelled structure of SARS-CoV-2 M protein with the objective of identifying some active phytochemicals as inhibitors. The entire study was carried out using different modules of Schrodinger Suite 2020-3. During the docking of the phytochemicals against the SARS-CoV-2 M protein, a compound, ZIN1722 from *Zingiber officinale* showed the best binding affinity with the receptor with a Glide Docking Score of −5.752 and Glide gscore of −5.789. In order to study the binding stability, the complex between the SARS-CoV-2 M protein and ZIN1722 was subjected to 50 ns Molecular Dynamics simulation using Desmond module of Schrodinger suite 2020-3, during which the receptor-ligand complex showed substantial stability after 32 ns of MD Simulation. The molecule ZIN1722 also showed promising results during ADME-Tox analysis performed using Swiss ADME and pkCSM. With all the findings of this extensive computational study, the compound ZIN1722 is proposed as a potential inhibitor to the SARS-CoV-2 M protein, which may subsequently prevent the immunosuppression mechanism in the human body during the SARS-CoV-2 virus infection. Further studies based on this work would pave the way towards the identification of an effective therapeutic regime for the treatment and management of SARS-CoV-2 infection in a precise and sustainable manner.

## Introduction

### Research Question

Owning to the massive global threat posed by Covid-19, discovery of novel and effective therapeutic strategies against the disease becomes the need of the hour (Nand et al., 2020). The present study was designed to computationally identify phytocompound(s) against Membrane Glycoprotein (M protein) of SARS-CoV-2 with a view to targeting the antagonistic effect M protein exerts on Type I Interferon induced anti-viral immunity in the host cell.

### The Disease and Its Causative

Since the first recorded case in November 2019 in Wuhan, China, Covid-19 has gone on to infect citizens in all the continents (Puttaswamy et al., 2020; Elekofehinti et al., 2021). The National Health Commission of China went on to confirm the human-to-human transmission of the disease in late January 2020, following which on 31 January, 2020, WHO pronounced COVID-19 as a ‘public health emergency of international concern (11 March 2020)’, and subsequently as a pandemic in March 2020 (Aouidate et al., 2021; Elfiky, 2021; Sridhar et al., 2021). As of 21 October, 2021, there have been a total of 241,886,635 recorded Covid-19 cases and 4,919,755 reported deaths (WHO Coronavirus (COVID-19) Dashboard | WHO Coronavirus (COVID-19) Dashboard with Vaccination Data). Owing to its virulence and high rate of infection, the Covid-19 virus, SARS-CoV-2, has caused large-scale damage, leading to global economic losses (Andrianov et al., 2020; Jiménez-Alberto et al., 2020; Saxena, 2020; Elekofehinti et al., 2021).

Belonging to the RNA virus family, Coronaviridae, SARS-CoV-2 is a Betacoronavirus (genus) like the member coronaviruses, SARS and MERS (Aouidate et al., 2021; Elfiky, 2021). Covid-19, the recently identified Coronavirus disease caused by SARS-CoV-2, is way more fatal to humans compared to the previously identified strains (Mohanta, 2020; Puttaswamy et al., 2020).

SARS-CoV-2 enters the host cells with the help of ACE2, Angiotensin-Converting Enzyme 2, which is found attached to the cell membrane of several organs in the human body. SARS-CoV-2 endocytoses its way into a host cell via means of interacting with the ACE2 receptor using its unique surface glycoprotein, spike protein. The virus then goes on to hijack the replication machinery of the host cell and produces a new set of viral particles ready to infect a new set of cells (Elekofehinti et al., 2021).

SARS-CoV-2 primarily affects the lungs. Additionally, the virus affects other organs such as intestine, kidneys, blood vessels among several others (Palmeira et al., 2020; Elekofehinti et al., 2021).

Infected people display a wide range of symptoms varying from person-to-person including fever, dyspnea, dry cough, loss of appetite, vomiting, loss of sense of taste and smell, which in certain cases progresses to pneumonia leading to acute respiratory distress syndrome (ARDS), multi-organ failure, usually followed by death (Gimeno et al., 2020; Puttaswamy et al., 2020; Elekofehinti et al., 2021). Aggressive inflammatory response to SARS-CoV-2 infection may result in Cytokine Storm which mediates widespread lung infection and is often followed by septic shock and multi-organ failure, making Cytokine Storm one of the major causes of fatality among the patients (Mohanta T. K. et al., 2020; Tay et al., 2020).

Coronaviruses classify as an extremely diverse group of enveloped viruses having positive-sense, single-stranded RNA (ssRNA), possessing a genome bearing 27–32 kilobases (Khan et al., 2020; Shamsi et al., 2020). The genome contains at least 6 ORFs which code for a minimum of 4 structural proteins and 16 non-structural proteins (Jiménez-Alberto et al., 2020).

The four structural proteins include Nucleocapsid (N) protein, Envelope (E) protein, Membrane (M) protein, and Spike (S) protein (Sridhar et al., 2021). These four proteins together form the envelope of SARS-CoV-2 and are responsible primarily for the assembly of the virus and subsequently budding. These proteins also modulate the host’s cellular response to the infection caused by the virus (Elekofehinti et al., 2021). Specifically, the N protein is responsible for the processing of the viral genome, and the S proteins are the components that polymerize to give coronaviruses its popular crown-like shape in addition to being the primary component facilitating the virus entry into the host cell by the process of receptor-mediated endocytosis (Nand et al., 2020; Elekofehinti et al., 2021). Further, the M protein, through its transmembrane domain, binds to the nucleocapsid of the virus and provides a shape to the virion. The E protein plays a crucial role in viral pathogenesis and the virus assembly and budding. Whereas, the 16 non-structural proteins essentially play crucial roles in the virus replication machinery (Khan et al., 2020).

The genome of SARS-CoV-2 produces a ∼800 kDa polypeptide as its transcription product which is proteolytically cleaved by enzymes, 3-Chymotrypsin-like protease (3CLpro) and Papain-like protease (PLpro). Cleavage by 3CLpro essentially leads to the formation of the various non-structural proteins mentioned prior. Hence, 3CLpro was identified as a crucial protein (Nand et al., 2020). Owing to their roles, these proteins were identified as drug targets, however, among these, S protein and 3CLpro, identify as the most popular drug targets (Nand et al., 2020).

### Therapeutics and Its Status

The dramatic unfolding of the pandemic has drawn global concern towards the development of therapeutic measures against the virus (Morgon et al., 2021). Various researchers worldwide have adopted various strategies towards finding an effective drug molecule. Most approaches include the use of data from preliminary *in vitro* studies and clinical trials, computational techniques, high-throughput screening and drug-repurposing (Puttaswamy et al., 2020; Wu et al., 2020). Using these approaches, several studies had suggested potential drug candidates, which include now popular names like Remdesivir, Oseltamivir, Tocilizumab, Ribavirin, Chloroquine, Hydroxychloroquine, among others (Gimeno et al., 2020; Al-Sehemi et al., 2021; Tazikeh-Lemeski et al., 2021). These are mostly antivirals, corticosteroids, antibiotics and immunoglobulins (Morgon et al., 2021). There are, however, several issues around these compounds. The efficacy of most of these remains controversial (Gimeno et al., 2020). Several of the repurposed drugs displayed potent results in *in vitro* studies but failed to show encouraging results in clinical trials (Gogoi et al., 2021). Various others have failed to specifically interact with the viral protease (Elekofehinti et al., 2021). All in all, the absence of a safe, effective, potent and definitive drug molecule so far greatly emphasizes need to find newer potential candidates (Palmeira et al., 2020; Gogoi et al., 2021; Morgon et al., 2021; Tazikeh-Lemeski et al., 2021).

Even with vaccines out now, it’ll be 2 years before citizens of the poorest countries gain access to their doses (Padma, 2021). Additionally, the dramatically evolving variants of the virus resulting from mutations and ineffectiveness of the proposed molecules towards these variants (Andrianov et al., 2020), point to the need for identifying novel therapeutic approaches.

### Membrane Protein

M protein of SARS-CoV-2 plays a crucial role in the virus assembly (Bhowmik et al., 2020). M protein is inherently a glycoprotein that is generally conserved across the genus of Betacoronaviruses, especially across SARS CoVs (Bianchi et al., 2020). Despite the crucial role played by membrane proteins, few drugs are being developed targeting them (Ita, 2021). To mediate membrane fusion, SARS-CoV-2 spike glycoprotein must be sequentially cleaved at its S1/S2 and S2’ cleavage sites. Furin can cleave the polybasic insertion (PRRAR) at the S1/S2 cleavage site of SARS-CoV-2. (Peacock et al., 2021).

Innate Immunity, which is the first line of defence in the host against viral organisms, is triggered *via* PAMPs (Pathogen-Associated Molecular Patterns), which also includes ssRNAs, leading to the production of Interferons (IFNs): Type I (α, β) and Type III. These Type I and Type III IFNs, through a series of signalling cascades, go on to facilitate the expression of Interferon Stimulated Genes (ISGs) which confer antiviral immunity to the host cell (Zheng et al., 2020). In such a scenario, encoding of IFN antagonists by the viruses appears to be a common strategy adopted by the viruses to evade the host’s antiviral immunity. In recent screening studies, it was found that the M protein of SARS-CoV-2, inhibited SeV (Sendai virus)-induced IFN-β gene activation. The SARS-CoV-2 infection causes declined production of Type I and Type III IFNs, coupled with lowered ISG response. Whereas, in severe cases of infection, this expression is strongly triggered throughout the course of the infection (Zheng et al., 2020). Nonetheless, it has been established that SARS-CoV-2 M protein is a Type I and Type III Interferon-antagonist. It is to note that this antagonism is achieved by negatively influencing the formation of a multiprotein complex, the RIG-I/MDA-5–MAVS–TRAF3–TBK1 signalosome and by the impairing of MAVS (Mitochondrial Antiviral Signaling Protein) activation (Zheng et al., 2020; Fu et al., 2021).

The Type I IFNs (IFN-Is) confer strong antiviral immunity to the host cells, hence the antagonistic strategies adopted by SARS-CoV-2 to evade the host immunity has been an intense matter of research. Additionally, it was found that IFN-Is facilitates the severity of the Cytokine Storms, which is a notably major complication observed in Covid-19, often leading to ARDS, followed by death (Mohanta TK. et al., 2020) (Schreiber, 2020).

### Significance of Computational Studies in Covid-19 Research

After identification of a potential drug target, the development of a drug molecule right from identification of potential ligands to procurement of a marketable drug, needless to say, is an extremely time-consuming and expensive process (Al-Sehemi et al., 2020; Tazikeh-Lemeski et al., 2021) which in case of the deadly SARS-CoV-2, is not the most feasible approach (Sridhar et al., 2021). *In silico* methods like Structure-Based Drug Design (SBDD) or Computer-Aided Drug Discovery (CADD) techniques prove to be extremely rapid, cost-effective, robust and reliable alternatives (Al-Sehemi et al., 2020, 2021; Gimeno et al., 2020; Tatar et al., 2021). CADD techniques have been vigorously used by researchers for the identification of lead molecules for various diseases so far, including Covid-19, and presented encouraging results (Gentile et al., 2020; Gimeno et al., 2020; Naik et al., 2020; Aouidate et al., 2021).

### Significance of Compounds From Medicinal Plants

Owing to the numerous side effects, including drug resistance, posed by synthetic drugs, recent drug discovery has seen a shift towards natural products as sources of drug molecules (Gogoi et al., 2021). Plants are rich sources of pharmacologically active or bioactive compounds possessing huge structural diversity (Naik et al., 2020; Puttaswamy et al., 2020; Gogoi et al., 2021). These bioactive small molecules are the plant’s secondary metabolites (Puttaswamy et al., 2020). Extensive *in silico* research has been conducted since the outbreak towards identifying phytocompounds possessing anti-Covid-19 properties. Some of the proposed candidates include curcumin, epi-catechin-gallate, kaempferol, catechin, quercetin, garlic essential oil, compounds from *Houttuynia cordata*, among several others (Puttaswamy et al., 2020; Thuy et al., 2020; Das et al., 2021).

### Summary of the Current Study

In the present study, 7,832 compounds from 32 medicinal plants of India possessing traditionally established linkage with pneumonia-like disease treatment, were curated. These phytocompounds were subsequently virtually screened, using CADD protocols, against a Homology Modelled structure of SARS-CoV-2 M protein. The ligands were subjected to Molecular Docking and subsequently to Molecular Dynamics simulation of 50 nanoseconds (ns), both of which were carried out in Schrödinger Suite 2020-3. The finally shortlisted ligands were evaluated for their ADMET properties. Following the experiments, the ligand, ZIN1722, from *Zingiber officinale*, emerged as the best binding ligand, indicating its potential anti-Covid-19 property.

## Materials and Methods

### Prediction of the 3D Structure of SARS-CoV-2 M Protein

Due to the absence of the structure of the SARS-CoV-2 M protein in Protein Data Bank, the 3D-structure of the protein was determined via Homology Modelling (Venselaar et al., 2010).

The sequence for the protein, encoded by gene M, was retrieved from UniProt SwissProt database (SwissProt accession number P0DTC5.1) (Boeckmann et al., 2003). The 222-amino acid-long protein was subjected to Homology Modelling using homology/ab initio hybrid online server BhageerathH+ (Jayaram et al., 2014).

The analysis of the quality of the predicted structure of M protein was performed using ProSA analysis (Wiederstein and Sippl, 2007) and subsequently, the Ramachandran plot was generated for the predicted structure (Ho et al., 2009) through ProCheck (Roman Laskowski et al., 1983).

### Preparation of the Receptor Target: SARS-CoV-2 M Protein

The predicted 3D structure of the SARS-CoV-2 M protein was processed and prepared for Molecular Docking in Protein Preparation Wizard of Epik module of Schrödinger Suite, 2020-3 (Paul et al., 2021). The similar binding sites and excess water molecules in the protein structure were removed, and the bond orders were refined. Using the Prime module of Schrodinger Suite, 2020-3, the missing chain atoms were added (Suganya et al., 2014). Further, the 3D structure of the protein was minimized using Optimized Potentials For Liquid Simulations-3 (OPLS3e) molecular force field with RMSD (Root Mean Square Deviation) of crystallographic heavy atoms which were kept at 0.3Å (Paul et al., 2021).

The structure was further subjected to active site analysis on AADS which is an Automated Active Site Identification server developed by the Supercomputing Facility for Bioinformatics and Computational Biology, IIT Delhi (Singh et al., 2011).

### Selection and Preparation of the Ligands

A ligand library of 7,832 compounds from 32 traditionally used medicinal plants of India was curated. To prepare the ligands for Molecular Docking and further studies, a series of activities were executed. The two-dimensional structures of the ligands were converted to their respective 3D counterparts using the LigPrep module of the Schrödinger Suite (Schrödinger, 2017). Further, to screen out the unfavourable molecules from the library, Lipinski’s rule of Drug Discovery was used as the criteria (Lipinski, 2004). Next, energy minimization and geometry optimization were performed for the 3D structures of the ligands, followed by desaltation and correction of chirality. The tautomeric and ionization states were generated between pH 6.8–7.2 using Epik module of Schrödinger suite 2020-3. The ligand structures were minimized using OPLS3e force field in Schrödinger Suite 2020-3 until a Root Mean Square Deviation (RMSD) value of 2.0Å was attained. The optimized ligands were docked against the selected receptor proteins.

### Molecular Docking

Molecular Docking was performed between the receptor and the optimized ligands in Glide module of Schrödinger Suite 2020-3 using the High Throughput Virtual Screening (HTVS) mode. Taking into consideration the Glide Docking Score, fifty ligands were shortlisted and further subjected to Extra Precision (XP) docking option of Glide (Wilmes et al., 2004; Schrödinger, 2017). Prior to performing molecular docking, grid box of the receptor protein structure was defined on the basis of the information of the predicted active site.

### Molecular Dynamics Simulation

The stability of the interactions within the docked complex constituted by SARS-CoV-2 M protein and the best-binding ligand was analyzed by performing Molecular Dynamic simulation of 50 ns (Schrödinger, 2017). The SARS-CoV-2 M Protein-ligand complex within the explicit solvent system with the OPLS3e force field was studied using the Desmond module of Schrödinger suite 2020-3. The atomic framework had been solvated with TIP3P crystallographic water particles with orthorhombic intermittent limit conditions for a 10 Å buffer region. The overlapping water molecules were eliminated and Na^+^ were added as counter ions to neutralize the entire framework of atoms. An ensemble (NPT) of Nose-Hoover thermostat and barostat was applied to maintain the constant temperature of 300 K and pressure of 1 bar in the system (Schrödinger, 2017). A hybrid energy minimization algorithm with 1,000 steps of steepest descent followed by conjugate gradient algorithms was used.

### ADME-Tox Analysis

To assess the safety and pharmacological competency of the shortlisted ligand, that is, to assess the absorption, distribution, metabolism, excretion and toxicity of the shortlisted ligand, ADME-Tox test of the ligand was performed. The ADME-Tox analysis was performed using two online tools, SwissADME and pkCSM, to encompass a pool of pharmacokinetic and medicinal chemistry properties (Pires et al., 2015; Daina et al., 2017).

• *S* ***wiss ADME***

SwissADME is a freely-available, user-friendly online tool which hosts numerous fast and robust predictive models for the evaluation of pharmacokinetics, physicochemical properties, drug-likeliness and medicinal chemistry friendliness.

• ***pkCSM***

pkCSM is an integrated, freely-available, user-friendly web server that utilises graph-based signatures to perform ADMET studies. These signatures encode distance patterns between the atoms which are in turn used to represent the small molecule as well as train the predictive models.

## Results

### Molecular Docking

The homology modelled-3D-structure of SARS-CoV-2 M protein was docked against the selected ligands using the High Throughput Virtual Screening (HTVS) and subsequently, Extra Precision (XP) modes of the Glide docking module of the Schrödinger Suite, version 2020-3. The docking score and other relevant information regarding the best twenty (20) docked complexes have been presented in Table 1. The results of the docking experiment presented a list of the best-binding ligands with respect to the receptor. With a Glide gscore of −5.789, ZIN1722, a compound from the root of *Zingiber officinale* (Ginger), emerged as the best-binding ligand.

**TABLE 1:** Docking score and ranking of the compounds evaluated through Glide Docking module.

Docking results	Title	Glide g score	Glide h-bond	Glide evdw	Glide ecoul	Glide erotb	Glide emodel	Glide energy	Glide einternal	Glide rmsd to input
Sl. No										
1	ZIN1722	−5.789	0	−16.754	−20.401	0.184	−49.137	−37.156	8.587	257.152
2	ZIN1693	−5.766	−0.138	−22.337	−23.704	0.297	−59.278	−46.041	10.482	258.095
3	ZIN1743	−5.766	−0.138	−22.337	−23.704	0.297	−59.278	−46.041	10.482	258.095
4	ZIN1756	−5.766	−0.138	−22.337	−23.704	0.297	−59.278	−46.041	10.482	258.095
5	ZIN1764	−5.766	−0.138	−22.337	−23.704	0.297	−59.278	−46.041	10.482	258.095
6	ZIN1785	−5.766	−0.138	−22.337	−23.704	0.297	−59.278	−46.041	10.482	258.095
7	ZIN1832	−5.613	0	−19.176	−18.007	0.171	−48.022	−37.183	9.35	259.004
8	ZIN1828	−5.587	0	−20.061	−17.752	0.171	−48.776	−37.813	8.361	259.037
9	ZIN1721	−5.583	−0.37	−26.386	−20.096	0.353	−65.374	−46.482	6.238	259.349
10	ZIN1742	−5.583	−0.37	−26.386	−20.096	0.353	−65.374	−46.482	6.238	259.349
11	ZIN1783	−5.583	−0.37	−26.386	−20.096	0.353	−65.374	−46.482	6.238	259.349
12	ZIN1824	−5.583	−0.37	−26.386	−20.096	0.353	−65.374	−46.482	6.238	259.349
13	ZIN1747	−5.476	−0.64	−28.754	−17.483	0.333	−61.77	−46.237	4.577	262.718
14	RF112	−5.39	−0.32	−1.974	−10.533	0	−16.927	−12.508	0.35	261.539
15	CEN53	−5.252	−0.32	−23.968	−18.418	0.399	−55.938	−42.386	5.911	260.935
16	PI542	−5.138	−0.16	−30.631	−14.72	0.711	−60.674	−45.351	4.286	254.177
17	COR7	−5.13	0	−20.714	−11.882	0.765	−39.732	−32.596	5.616	258.797
18	TUR45	−5.115	−0.238	−36.111	−7.935	0.921	−53.639	−44.046	7.621	257.545
19	ZIN1720	−5.103	−0.137	−22.318	−19.716	0.395	−52.334	−42.035	10.944	259.016
20	ZIN1754	−5.103	−0.137	−22.318	−19.716	0.395	−52.334	−42.035	10.944	259.016

**FIGURE 1:**
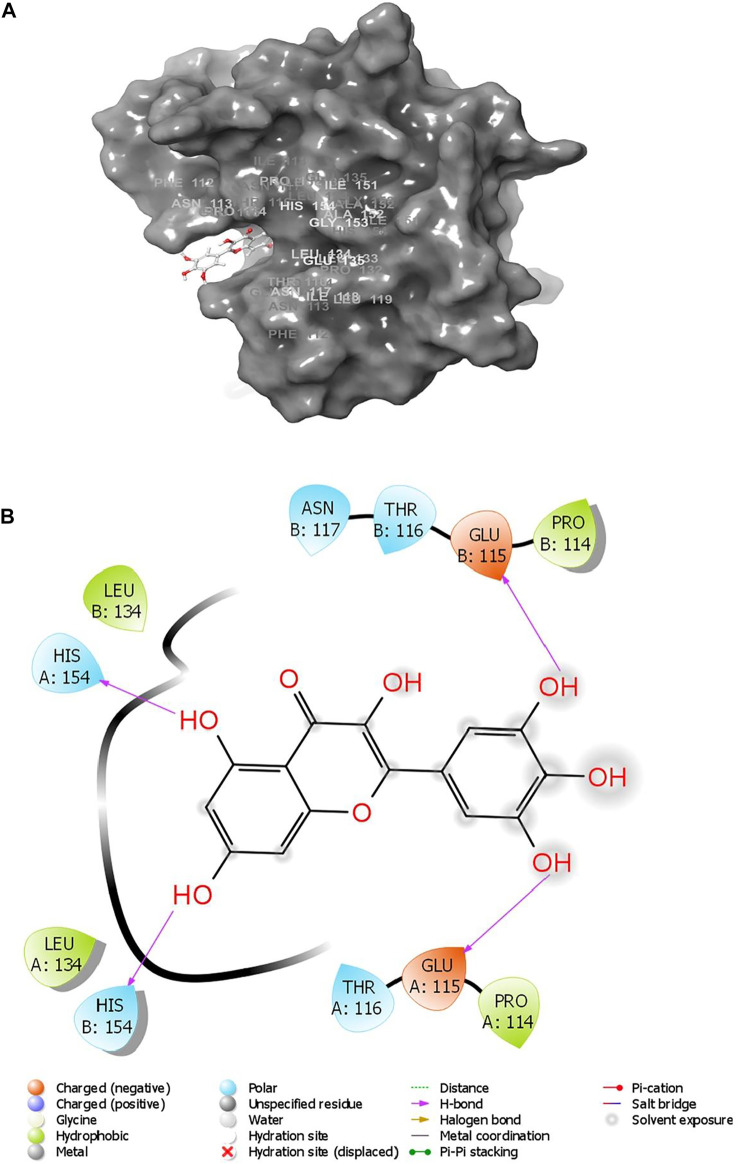
**(A)** Surface view of the interactions between SARS-CoV-2 M Protein bound to the ligand ZIN1722; **(B)** Detailed interaction diagram of SARS-CoV-2 M Protein with ZIN 1722. [before MD simulation].

Various interactions were observed between the compound, ZIN1722, and the structure of M protein. A total of four hydrogen bonds were noted. Glu115 and His154 of both the chains of the receptor have formed hydrogen bonds with the ligand. The interactions have been pictorially represented in Figures 1A,B. The compound, ZIN1722, hence, was considered for further study based on its best *in silico* affinity towards the receptor among all the ligands.

### Molecular Dynamics Simulation

**FIGURE 2:**
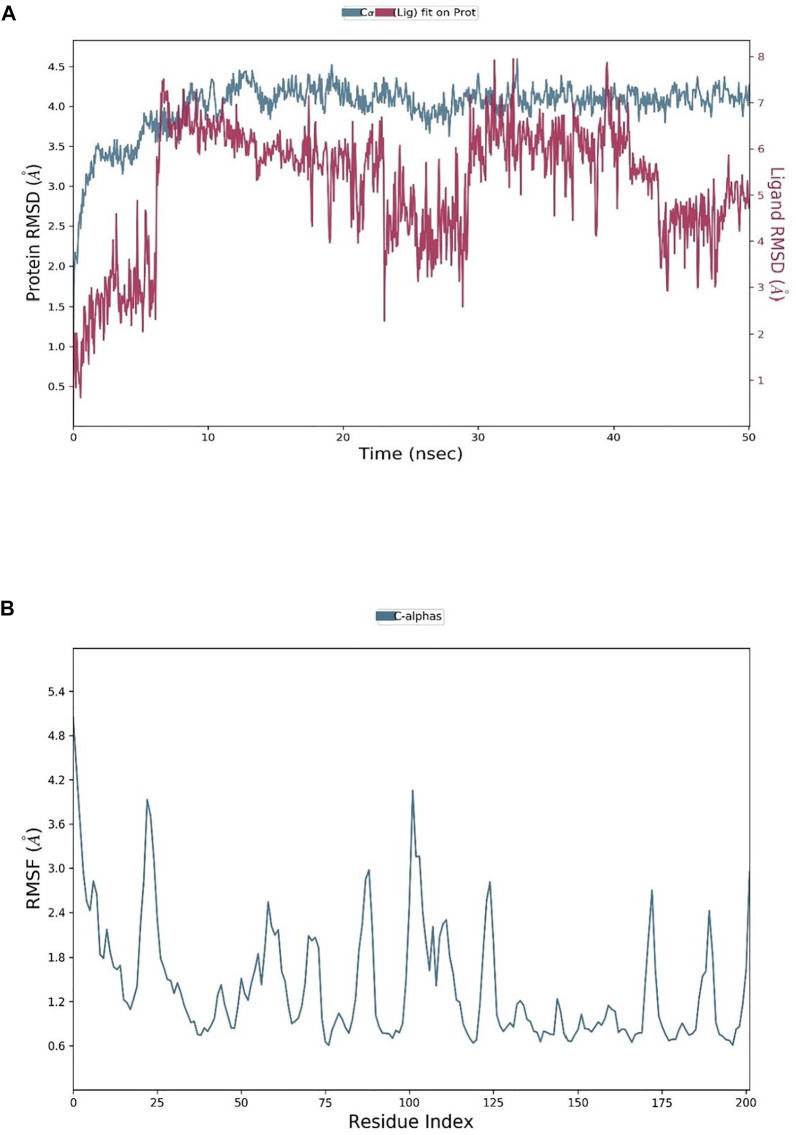
**(A)** The RMSD plot for SARS-CoV-2 M protein and ZIN1722 complex, where the RMSDs of Protein backbone and the Ligands were calculated separately throughout the MD trajectory of 50 ns; **(B)** RMSF plot of the Protein chain in Ligand bound state.

**FIGURE 3:**
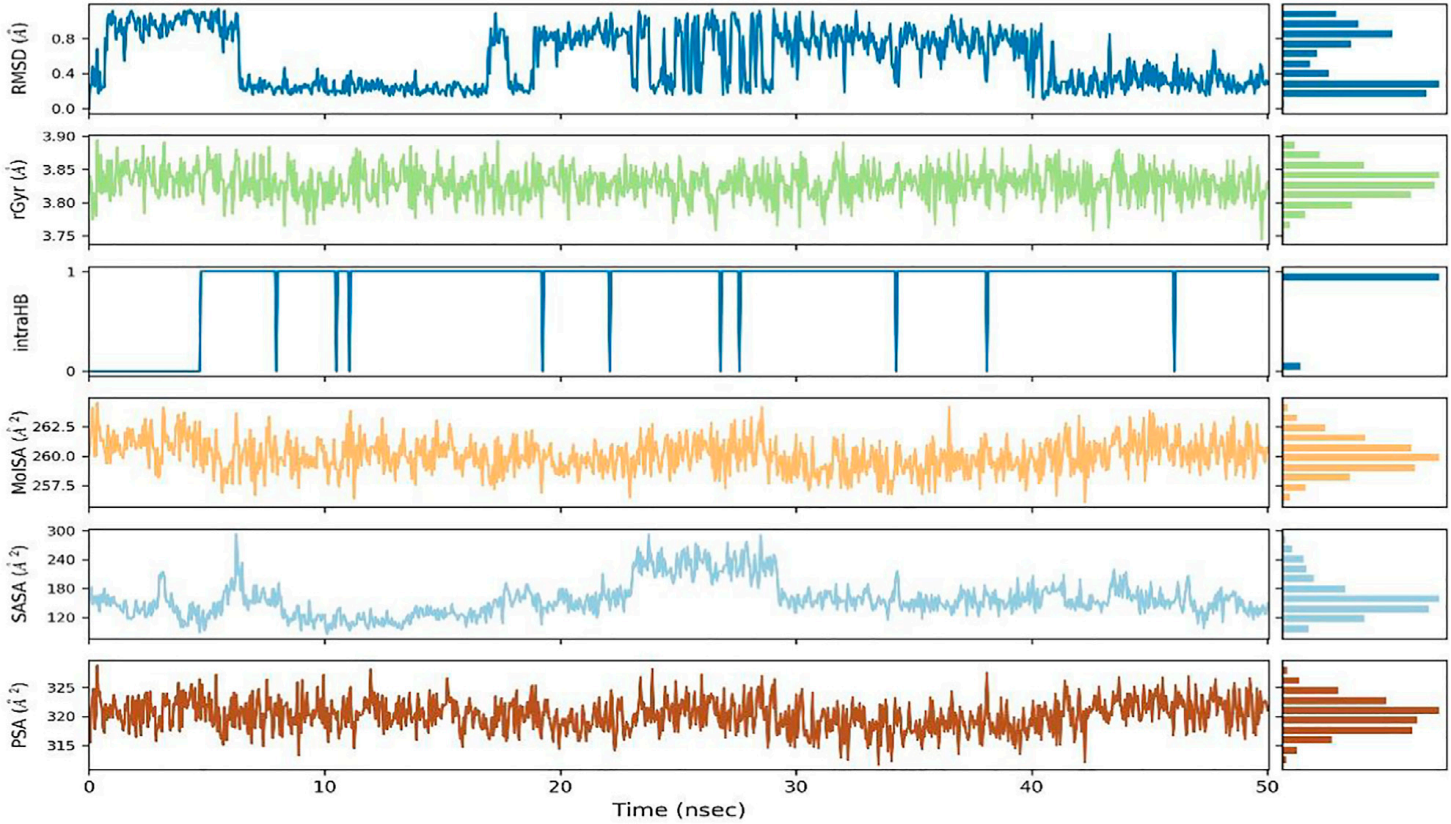
Ligand RMSD, Radius of Gyration (rGyr), Intramolecular Hydrogen Bond (intraHB), Molecular Surface Area (MolSA), Solvent Accessible Surface Area (SASA), Polar Surface Area (PSA) as calculated during the 50 ns of MD Simulation.

**FIGURE 4:**
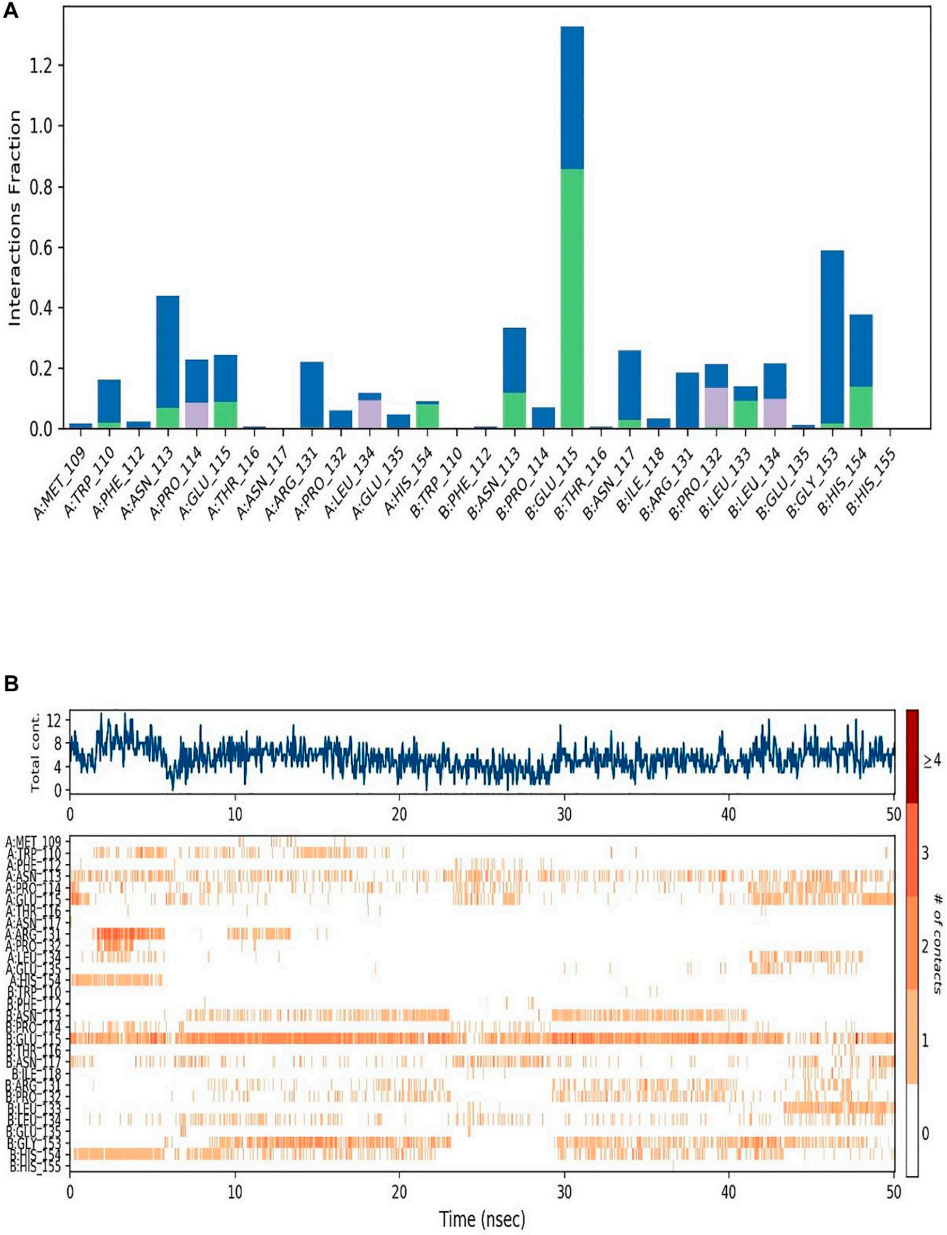
**(A)** Interactions between the SARS-CoV-2 M protein and the Ligand throughout the Simulation is depicted in bars. Different color signifies different interactions and contacts formed between the amino acids and the ligand atoms. **(B)** Graphical representation of the interactions and contacts throughout the Simulation.

To assess the stability of the interactions between ZIN1722 and the M protein structure, the docked complex was studied under Molecular Dynamics Simulation of 50 ns in Desmond software, Schrödinger Suite version 2020-3. The results of the Molecular Dynamics simulation study have been presented in Figures 2–4. In Figure 2A, the left *Y*-axis represents the RMSD evolution of the proteins, that is*,* the structural conformation of the proteins throughout the simulation and the right *Y*-axis represents the stability of the ligand with respect to the protein and the protein’s binding pocket. The Root Mean Square Deviation (RMSD) of the ligand, ZIN1722, bound to SARS-CoV-2 M Protein, was found to be stable after 32 ns of the simulation. Whereas, the RMSD of the receptor protein, bound to the ligand, was almost stable throughout the entire 50 ns of the simulation which signifies that the structural conformation of the receptor protein in the ligand-bound state was quite stable. The Ligand RMSD, Intramolecular Hydrogen Bonds, Radius of Gyration, Molecular Surface Area, Polar Surface Area and Solvent Accessible Surface Area of ZIN1722 in complex with the receptor protein was studied further during the 50 ns-long simulation and the resultant images are presented in Figure 3.

In the RMSF (Root Mean Square Fluctuations) local charges throughout the protein chain were characterized. The RMSF plots of the receptors bound to the selected ligands are placed in Figure 2B. The peaks in Figure 2B indicate the protein residues that fluctuated the most during the entire 50 ns-long simulation; the green-coloured vertical bars indicate the residues of the protein that interacted with the ligand. The fluctuations around amino acids no 25, 50, 100, 125 and 175 were recorded along with the N and C terminal amino acids as expected. As a rule, the N- and C-terminal regions of proteins fluctuate more than the rest of the residues, similarly, the structured regions like alpha helices and beta strands fluctuate less than the loop regions. Throughout the trajectory, mostly water bridges, Hydrogen bonds and Hydrophobic interactions were recorded between the receptor and the ligand. The amino acids Glu115, His154, Leu135 and Asn 113 had shown Hydrogen bonding with different atoms of the ligand ZIN1722. A hydrogen bond with Glu115 was formed and maintained over more than 80% of the total duration of the simulation.

A summary of the interactions between the receptor and the ligand throughout the MD simulation has been presented in Figures 4A,B.

### ADME-Tox Analysis

The pharmacokinetic properties and toxicity status of ZIN1722 were assessed using pkCSM and SWISS ADME web servers. The compound passed the Lipinski rule of five and Ghose filter which indicates that it may be a good lead compound. The absorption value of ZIN1722 predicted by pkCSM server is 65.4% whereas, the volume of distribution is 1.232 (log L/kg). Total clearance, which is an indicator of excretion value, is predicted to 0.526 (log ml/min/kg) whereas, oral rat acute toxicity (Rat LD50 for toxicity) is predicted to be 2.437 (mol/kg), which is in a quite acceptable range. The detailed report of the ADME-Tox study performed on both the aforementioned servers has been presented in Table 2 and Table 3. All in all, both the *in silico* ADME-Tox tests present encouraging results. Hence, the molecule, ZIN1722, may be considered for *in vitro* validation and subsequent studies.

**TABLE 2:** Summary of the ADME-Tox results performed using SWISS-ADME server.

Compound	Oral bioavailability							Pharmacokinetic properties	LogKp (skin permeation)	Water solubility	Lipinski/Ghose/Veber (pass(Y)/ Fail (N))
	MW	cLogP	HBA	HBD	RB	Tpsa (Å^2^)	B-score				
ZIN17222	317.23 g/mol	0.97	8	5	1	154.42	0.11	GI Absorption Low	−7.40 cm/s	Soluble	Y/Y/N

MW: molecular weight; cLogP: consensus lipophilicity score; HBA: H-bond Acceptor; HBD: H-bond donor; RB: No. of rotatable bonds; PSA: polar surface area; B-score: Bioavailability score; Lipinski/ Ghose/Veber: Rules of Drug Discovery.

**TABLE 3:** Summary of the ADME-Tox study performed using pkCSM server.

Property	Model name	Predicted value	Unit
**Absorption**	Water solubility	**−2.98**	Numeric (log mol/L)
	Caco2 permeability	**0.234**	Numeric (log Papp in 10^-6^ cm/s)
	Intestinal absorption (human)	**65.4**	Numeric (% Absorbed)
	Skin Permeability	**−2.735**	Numeric (log Kp)
	P-glycoprotein substrate	**Yes**	Categorical (Yes/No)
	P-glycoprotein I inhibitor	**No**	Categorical (Yes/No)
	P-glycoprotein II inhibitor	**No**	Categorical (Yes/No)
**Distribution**	VDss (human)	**1.232**	Numeric (log L/kg)
	Fraction unbound (human)	**0.196**	Numeric (Fu)
	BBB permeability	**−1.135**	Numeric (log BB)
	CNS permeability	**−3.522**	Numeric (log PS)
**Metabolism**	CYP2D6 substrate	**No**	Categorical (Yes/No)
	CYP3A4 substrate	**No**	Categorical (Yes/No)
	CYP1A2 inhibitor	**Yes**	Categorical (Yes/No)
	CYP2C19 inhibitor	**No**	Categorical (Yes/No)
	CYP2C9 inhibitor	**No**	Categorical (Yes/No)
	CYP2D6 inhibitor	**No**	Categorical (Yes/No)
	CYP3A4 inhibitor	**No**	Categorical (Yes/No)
**Excretion**	Total Clearance	**0.526**	Numeric (log ml/min/kg)
	Renal OCT2 substrate	**No**	Categorical (Yes/No)
**Toxicity**	AMES toxicity	**No**	Categorical (Yes/No)
	Max. tolerated dose (human)	**0.535**	Numeric (log mg/kg/day)
	hERG I inhibitor	**No**	Categorical (Yes/No)
	hERG II inhibitor	**No**	Categorical (Yes/No)
	Oral Rat Acute Toxicity (LD50)	**2.437**	Numeric (mol/kg)
	Oral Rat Chronic Toxicity (LOAEL)	**2.28**	Numeric (log mg/kg_bw/day)
	Hepatotoxicity	**No**	Categorical (Yes/No)
	Skin Sensitization	**No**	Categorical (Yes/No)
	*T.Pyriformis* toxicity	**0.303**	Numeric (log ug/L)
	Minnow toxicity	**4.244**	Numeric (log mM)

The phytocompound, ZIN1722, bears the common name Myricetin. Its IUPAC name is 5,7-Dihydroxy-4-Oxo-2-(3,4,5-Trihydroxyphenyl)-4H-Chromen-3-Olate. It is obtained from the root of *Zingiber officinale*, commonly known as Ginger. The PubChem Compound ID (CID) is 25201643 (Kim et al., 2021).

## Discussion

Ever since its outbreak in 2019, Covid-19 has caused massive global damage in all spheres. Needless to say, in the absence of a single safe, effective and definite treatment measure and the rapidly mutating strains of the virus, SARS-CoV-2, the discovery of new and potent drug candidates as well as novel therapeutic approaches, become the need of the hour (Andrianov et al., 2020; Nand et al., 2020; Palmeira et al., 2020; Gogoi et al., 2021; Morgon et al., 2021; Padma, 2021; Tazikeh-Lemeski et al., 2021). The present study was designed with a view to targeting the less popular drug target of SARS-CoV-2, Membrane Glycoprotein, also known as M Protein. M protein plays a critical role in various functions of the virus, mainly in virus assembly (Bhowmik et al., 2020). However, this study was designed targeting another functionally significant role of M protein in infecting a host. M protein facilitates evasion of the host’s immune system by means of rendering a declined production of Type I and Type III Interferon by suppressing the expression of Interferon Stimulated Genes (ISGs) (Schreiber, 2020; Zheng et al., 2020; Fu et al., 2021). Type I IFN confers strong anti-viral immunity to the host, hence, inhibiting M proteins which inhibit the production of Type I IFN, may hinder disease progression in the infected individuals. In the present study, 7,832 phytocompounds from 32 medicinal plants of India were virtually screened, resulting in the identification of compound ZIN1722 as the compound with the best binding affinity towards the receptor protein, M protein, whose structure was predicted through Homology Modelling. To study the stability of the interactions formed between ZIN1722 and the M protein, the complex was subjected to a 50 ns-long Molecular Dynamics Simulation. Throughout the trajectory, it was found that the receptor-ligand complex displayed considerable stability and hence, it can be concluded that the ligand, ZIN1722, can be considered as a compound possessing encouraging affinity towards SARS-CoV-2 M protein. Further, the safety and pharmacological competency of the phytocompound was assessed through extensive pharmacokinetic profiling performed on SwissADME and pkCSM webservers. The compound passed the Lipinski rule of five and Ghose filter, and moderate-to-excellent values of most of the pharmacokinetic parameters were assessed. Overall, results of the *in silico* ADME-Tox analysis indicate encouraging scope of the compound, ZIN1722, as a drug candidate. Based on these findings, ZIN1722 may be considered for further *in vitro* and *in vivo* validation as a novel anti-SARS-CoV-2 compound (Daina et al., 2017).

The phytocompound, ZIN1722, also known as Myricetin [PubChem Compound ID (CID) is 25201643; IUPAC name: 5,7-Dihydroxy-4-Oxo-2-(3,4,5-Trihydroxyphenyl)-4H-Chromen-3-Olate] is a flavonoid found mainly in the roots of Ginger (*Zingiber officinale*) (Yu et al., 2012; Song et al., 2021). Myricetin is also found in plants of many families and distributed in many foods like berries, fruits, vegetables, tea, etc.

From the current study, it was found that ZIN1722 or Myricetin has a considerable affinity towards SARS-CoV-2 M protein which is sufficient for inhibiting the activity of M protein which may disrupt the process of evasion of host antiviral immunity by means of inhibiting the declined production of Type I IFN and subsequently, lowered expression of ISGs. Inhibition of M protein is also expected to disrupt the process of viral assembly. Conclusively, inhibition of SARS-CoV-2 M protein by Myricetin is expected to mitigate viral infection in the infected individual, thereby, opening up novel therapeutic approaches to tackling the Covid-19 virus.

Myricetin has been previously reported to possess anti-viral, anti-bacterial, anti-tumour, anti-inflammatory activities. The compound is also known to be protective against cardiovascular diseases, neurological damage and potential liver injury (Semwal et al., 2016; Song et al., 2021).

In previous studies, Myricetin has also been reported to show high binding affinity towards ACE2 receptor, indicating the compound’s potential inhibitory activity towards viral entry into host cell during SARS-CoV-2 infection. Additionally, Myricetin has also been reported to possess inhibitory activity towards SARS-CoV helicase which supports the potential of this molecule in combating diseases caused by coronaviruses, including Covid-19 (Yu et al., 2012).

The findings of the current study are encouraging and align with previous research findings. The compound, Myricetin, identified through this *in silico* study as a potent anti-SARS-CoV-2 agent, possesses hopeful drug-like properties which can be further validated through *in vitro* and *in vivo* assays, and subsequent clinical studies, thereby, paving way for the identification of a potential therapeutic agent against Covid-19.

## Conclusion

Two years into the Covid-19 pandemic with countless fatalities, damage of varied kinds and degrees worldwide, no definitive drug molecule or treatment strategy, quest for an effective, potent and novel therapeutic approach against the disease becomes the need of the hour. The current study is a computational approach towards identifying a novel molecule against the causative of Covid-19, SARS-CoV-2. Digressing from the popularly studied receptor targets, the current study focuses on the immunological perspective of the infection, involving the SARS-CoV-2 Membrane Glycoprotein, M protein. In addition to playing a role in virus assembly, SARS-CoV-2 M protein has been reported to cause a declined production of Type I Interferon and a downregulated expression of the Interferon Stimulated Genes (ISGs), thereby facilitating evasion of the virus by the host innate immunity and subsequently, rendering a decreased anti-viral response of the host immune system towards the infection. Keeping in view this recent, valuable finding, this study was designed to identify inhibitory molecule(s) towards the SARS-CoV-2 M protein. In this study, 7,832 compounds from 32 medicinal plants of India traditionally known to be used in the treatment of pneumonia-like diseases, were virtually screened against a Homology Modelled structure of SARS-CoV-2 M protein. The best-binding ligand from the Molecular Docking experiment was further studied for its stability with the receptor through Molecular Dynamics simulation study, and for its pharmacokinetic profile through ADME-Tox study. The phytocompound, ZIN1722, that is, Myricetin was identified from this study as a potent SARS-CoV-2 M protein inhibitor. The findings of this study align with the findings of previously reported studies. The findings of this study are preliminary but highly encouraging, hence, further evaluation of the potency and efficacy of the phytocompound through *in vitro* and *in vivo* validation assays, and subsequent clinical studies, present hopeful scope towards the discovery of a novel therapeutic molecule against SARS-CoV-2 in the future (WHO Coronavirus Dashboard).

## Data Availability

The datasets presented in this study can be found in online repositories. The names of the repository/repositories and accession number(s) can be found in the article/Supplementary Material.
