# Supplementary material for: Plant Source Derived Compound Exhibited In Silico Inhibition of Membrane Glycoprotein In SARS-CoV-2: Paving the Way to Discover a New Class of Compound For Treatment of COVID-19
Source: Front Pharmacol. 2022 Apr 7;13:805344. doi: 10.3389/fphar.2022.805344 (PMC9022603; doi:10.3389/fphar.2022.805344)
Supplement: Supplementary file 1 [file Table1.DOCX]

| **Sl No.** | **Name of the plants** |
| --- | --- |
| **1** | ***Curcuma longa*** |
| **2** | ***Piper nigrum*** |
| **3** | ***Zingiber officinale*** |
| **4** | ***Withania somnifera*** |
| **5** | ***Alstonia scholaris*** |
| **6** | ***Rauwolfia serpentina*** |
| **7** | ***Achyranthes aspera*** |
| **8** | ***Justicia adhatoda*** |
| **9** | ***Calotropis procera.*** |
| **10** | ***Carica papaya*** |
| **11** | ***Centella asiatica*** |
| **12** | ***Cyperus rotundus*** |
| **13** | ***Colocasia esculenta*** |
| **14** | ***Terminalia bellerica*** |
| **15** | ***Argyreia nervosa*** |
| **16** | ***Murraya koenigii*** |
| **17** | ***Justicia gendarussa*** |
| **18** | ***Tinospora cordifolia*** |
| **19** | ***Cydonia oblonga*** |
| **20** | ***Momordica charantia*** |
| **21** | ***Moringa oleifera*** |
| **22** | ***Nigella sativa*** |
| **23** | ***Andrographis paniculate*** |
| **24** | ***Cordia dichotoma*** |
| **25** | ***Zizyphus vulgaris*** |
| **26** | ***Rubia cordifolia*** |
| **27** | ***Crocus sativus*** |
| **28** | ***Nigella sativa*** |
| **29** | ***Carica papaya*** |
| **30** | ***Dalbergia sisso*** |
| **31** | ***Hibiscus mutabilis*** |
| **32** | ***Centella japonica*** |

**Table S1: List of plants taken for the current research work**
